# Literature Review of Algorithms for Classifying Dementia and Mild Cognitive Impairment

**DOI:** 10.1093/geroni/igaf122.2131

**Published:** 2025-12-31

**Authors:** Tsai-Chin Cho, Chihua Li, Kelvin Zhang, Wenjie Cai, Kenneth Langa, Lindsay Kobayashi, Alden Gross

**Affiliations:** University of Michigan School of Public Health, Ann Arbor, Michigan, United States; University of Macau, Macao, Macao, Macau; University of Michigan School of Public Health, Ann Arbor, Michigan, United States; University of Michigan School of Public Health, Ann Arbor, Michigan, United States; University of Michigan School of Public Health, Ann Arbor, Michigan, United States; Johns Hopkins University Bloomberg School of Public Health, Baltimore, Maryland, United States

## Abstract

Demand is increasing for research algorithms that classify mild cognitive impairment and Alzheimer’s disease and related dementias (AD/ADRD) based on a set of rules and data features. Rules may include well-established criteria, such as the NINCDS-ADRDA Alzheimer’s Criteria, Diagnostic and Statistical Manual of Mental Disorders (DSM), Clinical Dementia Rating (CDR), or National Institute on Aging and Alzheimer’s Association (NIA-AA) framework, as well as ad hoc machine learning methods. Data used in algorithms may contain one or multiple features, such as cognitive performance scores, functional performance scores, informant reports, or brain imaging data. Despite the availability of numerous algorithms, there remains a lack of guidance on the rubrics to evaluate dementia classification algorithms built upon clinical or observational data and validated by different gold standards. We thus conducted a systematic review and meta-analysis of existing dementia algorithms to summarize and compare their construction. We identified 4,565 published articles from January 01, 2013, to February 28, 2025 from PubMed and PsycINFO. After screening abstracts and full text, 451 articles were included. Existing algorithms relied on similar data sources: 48.8% leveraged the clinical and imaging data from the Alzheimer’s Disease Neuroimaging Initiative (ADNI). Almost all articles evaluated algorithmic criterion validity against a gold standard, most commonly clinical or consensus diagnosis (67.9% of algorithms). This critical appraisal of algorithms for distinguishing dementia and mild cognitive impairment from neurocognitively healthy individuals in published studies will help guide future investigation into developing and implementing algorithms of dementia classification.

